# White Light Spectroscopy for Sampling-Free Bacterial Contamination Detection During CAR T-Cells Production: Towards an On-Line and Real-Time System

**DOI:** 10.3390/bios15080512

**Published:** 2025-08-06

**Authors:** Bruno Wacogne, Naïs Vaccari, Claudia Koubevi, Charles-Louis Azzopardi, Bilal Karib, Alain Rouleau, Annie Frelet-Barrand

**Affiliations:** 1 CNRS, Institut FEMTO-ST, Université Marie et Louis Pasteur, 25000 Besançon, France; nais.vaccari@hotmail.com (N.V.); claudiakoubevi@yahoo.fr (C.K.); cl.azzopardi@femto-st.fr (C.-L.A.); bilal.karib@femto-st.fr (B.K.); alain.rouleau@femto-st.fr (A.R.); annie.frelet-barrand@femto-st.fr (A.F.-B.); 2 Centre d’Investigation Clinique, Centre Hospitalier Universitaire de Besançon, INSERM CIC 1431, 25030 Besançon, France

**Keywords:** CAR T-cell, white light spectroscopy, contamination detection, advanced therapy medicinal product, quality control

## Abstract

Advanced therapy medicinal products (ATMPs), especially effective against cancer, remain costly due to their reliance on genetically modified T cells. Contamination during production is a major concern, as traditional quality control methods involve samplings, which can themselves introduce contaminants. It is therefore necessary to develop methods for detecting contamination without sampling and, if possible, in real time. In this article, we present a white light spectroscopy method that makes this possible. It is based on shape analysis of the absorption spectrum, which evolves from an approximately Gaussian shape to a shape modified by the 1/λ component of bacterial absorption spectra when contamination develops. A warning value based on this shape descriptor is proposed. It is demonstrated that a few hours are sufficient to detect contamination and trigger an alarm to quickly stop the production. This time-saving should reduce the cost of these new drugs, making them accessible to as many people as possible. This method can be used regardless of the type of contaminants, provided that the shape of their absorption spectrum is sufficiently different from that of pure T cells so that the shape descriptor is efficient.

## 1. Introduction

Mammalian cell culture is one of the pillars of the life sciences. It supports fundamental and applied research, particularly the production of advanced therapy medicinal products. In all cases, cells are cultivated in specific media to increase their quantity and functionality. However, contamination could occur during this process and lead to the termination of cell cultures. Therefore, early, rapid, and sensitive quality control and pathogen detection strategies are crucial throughout the production process. The goal is to monitor production in real time and stop cell cultivation as soon as bacteria are detected. This could reduce production costs and make these promising medicines more widely available. More generally, this type of detection is one of the most challenging aspects in the fields of human health and the agri-food industry [1]. Quality controls are required during industrial drug and biosimilar production processes. Numerous physics-, biology-, and chemistry-based techniques have been developed to detect, identify, and enumerate bacteria [2,3].

The polymerase chain reaction (PCR), which is based on DNA amplification, is currently the gold standard for detecting bacteria. Quantitative PCR (QPCR) can quantify targeted pathogens [4]. However, PCR is highly specific to the target DNA of a given microorganism. More recent techniques based on molecular biotechnology are also employed for rapid, real-time, sensitive, and specific detection. These techniques include nucleic acid amplification tests, real-time polymerase chain reaction (RT-PCR), and loop-mediated isothermal amplification (LAMP) [5]. Methods such as the plate culture approach are still used because they are effective and cost-effective; however, they are time-consuming and have limited sensitivity. Other techniques are based on growth to make detection possible, but these are reserved for cultivable bacteria. These techniques measure different changes in gas to detect bacteria in blood for sepsis diagnosis [6], such as charged ionic metabolites measured by impedance and adenosine triphosphate (ATP). However, filtration is required to distinguish bacteria from other sources. Other techniques measure temperature by microcalorimetry and optical density at specific wavelengths [7]. The sensitivity of each method depends on the microorganism. Although a variety of chromogenic and fluorogenic culture media have been developed for the selective isolation and differentiation of Gram-positive (G+) and Gram-negative (G−) bacteria, they have longer detection times and limited sensitivity [8,9]. Fluorescence enables more sensitive and accurate techniques, as well as high-speed and rapid techniques that allow for simultaneous detection without the need for bacterial growth [10]. However, it requires the labeling of bacteria with fluorophores. Recently, new techniques have emerged, such as metal–organic frameworks (MOFs) and molecularly imprinted polymers (MIPs). This combination of materials results in a composite that significantly improves the sensitivity, selectivity, antibacterial efficiency, and environmental friendliness of microbial detection [11].

Other detection techniques include immunological assays, such as ELISA. These tests aim to detect a specific molecule using a labeled capturing agent specific to one target. While these methods have a short response time, they are less sensitive than PCR, and they are difficult to apply to unknown samples due to their specificity. Traditional methods are often complex, time-consuming, and labor-intensive. Alternative, innovative techniques based on the recognition of ligands and bacteria have been developed. These techniques enable the detection of bacteria without the need for cultures, allowing for faster detection and improved sensitivity and specificity. They also allow for automation and the creation of cost-effective devices. Ligands can be classical antibodies, aptamers [12], bacteriophages [13], antimicrobial peptides (AMPs) derived from ATP or ADP, or fluorogenic RNA-cleaving DNAzymes. This enables discrimination between bacteria and detection at very low concentrations. These techniques can be used with optical methods, such as Raman spectroscopy [14], or acoustic techniques, such as QCM (quartz crystal microbalance) [15], in bulk systems or on microfluidic chips. These methods enable label-free detection of bacteria and allow for online, real-time measurements as well as integration into biosensors [16]. Advancements in nanomaterials and biosensors enhance their sensitivity and specificity [17]. Techniques based on microfluidics (e.g., acoustophoresis and microdroplets), mass spectrometry, and microscopy are also used [18,19]. However, these techniques can be expensive and require expertise. In recent years, various commercial systems for detecting bacteria have emerged. These systems use either oxygen-coupled fluorescence compounds (BACTEC, Becton Dickinson [20]) or pH changes due to CO_2_ emitted by bacteria (BactAlert^®^, BioMérieux [21]).

All of the above techniques require culturing steps prior to analysis in order to increase bacteria concentration. These techniques also require sampling, which breaks the sterility of closed cultivation systems. They also require adding compounds and/or fluorophores to increase sensitivity. These requirements highlight the need for rapid, effective, reproducible, label-free, cost-effective, sampling-less, real-time, automatable techniques for detecting bacteria without prior identification. Currently, no technique fulfills all these criteria. In recent years, white light spectroscopy has proven to be a powerful technique for monitoring and counting mammalian cells and bacteria, including those of the ESKAPEE group [22,23,24,25]. This group includes highly virulent, antibiotic-resistant bacteria that cause hospital-acquired diseases. These bacteria include both Gram-positive and Gram-negative types [26]. Both B- and T-type lymphocytes, as well as primary T-cells isolated from healthy donors, can be monitored [27]. Furthermore, this technique can be easily integrated into a contactless system for real-time, noninvasive measurements. These recent publications prove the concept of using white light spectroscopy in CAR-T cell production. They also describe how this method can easily be integrated into a real-time, sampling-free device.

This paper describes using white light spectroscopy to detect bacterial contamination during mammalian cell cultivation monitoring. Contamination was induced using *Escherichia coli*, a well-known bacterium, but this method could be applied to other ESKAPEE group bacteria and possibly other contaminants. Mathematical descriptions of the absorption spectra of both species enable cell and bacterial concentrations to be measured simultaneously. Section 2 describes the materials and methods employed in this study. Section 3, “Experimental Results,” is divided into two parts. First, it proposes a spectroscopic marker for detecting contamination in mammalian cell cultures. This marker is based on analyzing the shapes of absorption spectra and, more precisely, how they change when contamination develops. A warning value is defined that triggers an alert when contamination is detected. Using synthetic spectra obtained from experimental spectra of CEM-C1 T-cell lines and bacteria, the minimum detectable concentration of bacteria can be calculated for various cell concentrations and for bacteria from the ESKAPEE group. Second, the marker detects contamination in a cell culture infected with *E. coli*. The evolution of the warning value during the development of contamination was clearly observed. Section 4 discusses the experimental results, the position of this work in relation to others, and the real-time, sampling-less possibilities offered by white light spectroscopy. Section 5 draws conclusions.

## 2. Materials and Methods

The objective of the present work was to perform bacterial contamination of mammalian cell cultures using the well-known bacterium *Escherichia coli* and follow these contaminations by spectral measurements using white light spectroscopy.

### 2.1. Mammalian Cell Culture

T lymphoblasts (CEM-C1 cells, ATCC^®^ CRL-2265^TM^) were provided by the French Blood Agency (EFS BFC, Besançon, France). The cells were grown in a phenol red-free RPMI-1640 medium supplemented with 25 mM HEPES, 10% heat-inactivated fetal bovine serum (FBS), and 1% penicillin (10 kU.mL^−1^)/streptomycin (10 mg.mL^−1^). The cells were maintained at 37 °C in a humidified atmosphere containing 5% CO_2_. Table 1 summarizes reagents, suppliers, and references for cell culture.

### 2.2. Bacteria Culture

The *E. coli* bacteria (strain 18265017, Fischer Scientific™, Illkirch, France) were stored long-term at −80 °C in LB medium/30% glycerol (*v*/*v*). They were thawed on a TSA gelose and incubated overnight. Precultures were made with 4 different clones in 10 mL in the same RPMI medium used for cells but without antibiotics (RPMI-atb) and incubated for at least 20 h at 37 °C, 200 rpm. Then, they were centrifuged at 7180 g for 10 min at RT, and DO was measured at a wavelength of 600 nm using a spectrophotometer (Biowave DNA, Biochrom Ltd., Cambridge, UK). Afterwards, the pellets were resuspended in an appropriate volume of RPMI-atb to adjust bacteria to a concentration of 1 × 10^7^ bact.mL^−1^.

Suspensions of bacteria were diluted in 0.85% NaCl at dilutions allowing bacteria measurements. A volume of 54.3 µL was plated on a Petri dish containing TSB medium with an automatic seeder (Spiral Platter Eddy Jet, I&L Biosystems, Konigswinter, Germany). The plates were incubated overnight before performing a manual enumeration following the manufacturer’s instructions. Table 2 summarizes reagents, suppliers, and references for bacteria culture.

### 2.3. Bacterial Contamination of Mammalian Cell Experiments

In total, 6 different experiments have been performed during 3 experimental sessions. For each session one control cuvette with 5 × 10^5^ cell.mL^−1^ was used as a control. For each experiment, two mixtures of the CEM cells and bacteria were prepared in duplicates with the following species concentrations in RPMI media without antibiotics:

- CEM at 5 × 10^5^ cell.mL^−1^ and *E. coli* at 1 × 10^6^ bact.mL^−1^
- CEM at 5 × 10^2^ cell.mL^−1^ and *E. coli* at 1 × 10^2^ bact.mL^−1^

Prior to the preparation of the mixtures, the species’ concentrations were measured optically using the Luna counter for mammalian cells and the optical density at 600 nm for bacteria. Then, mixtures were incubated within an incubator at 37 °C during 11 h.

### 2.4. Spectral Measurements

We performed spectral measurements of CEM suspensions with and without bacteria using the experimental setup described in [24,26]. Our spectroscopic system consisted of a light source connected to a cuvette holder via optical fibers. After propagating through the spectroscopic cuvette, the light was transmitted to the spectrophotometer for spectral acquisition.

Before each measurement, the reference spectrum was acquired using only RPMI-atb. Cell suspensions with or without bacteria were homogenized with several gentle inversions before each spectroscopic measurement. Measurements were performed every 30 min. The spectra were recorded in transmission in the wavelength range of 177–892 nm with a step size of 0.22 nm using OceanView software (see Table 3). Each experiment lasted 11 h. Spectra were acquired hourly, except during session 1, when they were recorded twice an hour between 4 and 11 h. Table 3 summarizes the equipment, suppliers, and references for the spectral measurements.

### 2.5. Spectral Data Processing

A total of 129 spectra were acquired during the six experiments, as detailed in Table 4. The transmission data were converted to absorption, and all calculations were performed using MATLAB™ software (version number R2020b; MATLAB™, USA; supplier: Meudon, France). Only wavelengths between 350 and 850 nm, for which the signal-to-noise ratio was high enough, were considered. As previously mentioned, artifacts due to the energetic emission peaks of the deuterium lamp were numerically removed [24]. The additional signal at 410 nm due to the varying FSB concentration was also removed numerically, as explained in [26]. Absorption spectra of neutral densities (Thorlabs, USA; Maisons Laffitte, France; part numbers NE05B and NE10B) were regularly recorded and compared to the supplier’s data to ensure correct absorption spectrum measurements. Only spectra containing useful information within the considered spectral range were kept; these spectra had an absorption range of 3% to 94% at a wavelength of 600 nm.

### 2.6. Synthetic Spectra Mimicking CEM-C1 Contaminated with ESKAPEE Bacteria

ESKAPEE is an acronym that refers to a model group of model bacteria involved in nosocomial diseases: *Escherichia coli*, *Staphylococcus aureus*, *Klebsiella pneumoniae*, *Acinetobacter baumannii*, *Pseudomonas aeruginosa*, *Enterococcus faecium*, and *Enterobacter cloacae*.

Synthetic spectra are artificial yet realistic data sets generated by algorithms or simulations rather than collected from real-world events. This type of data is used more and more in machine learning, AI development, testing, and privacy-sensitive environments. The goal is to produce data that preserves the key characteristics of the original data. In this study, synthetic spectra were computed using a spectral composition equation derived from the law of optical densities additivity (Equation (2) in [24]). This equation is obtained as follows: Equation (1) shows the law of additivity of optical densities for a mixture of “n” elements.

(1) $${OD}_{Mixture}=\sum_{i=1}^{n}{OD}_{i}$$

Here, ${OD}_{Mixture}$ is the optical density of the mixture and ${OD}_{i}$ the optical density of element “i”. Using the relationship between optical densities and transmittance yields the following:

(2) $${log}_{10}\left(T_{Mixture}\right)=\;\sum_{i=1}^{n}{log}_{10}T_{i}=\;{log}_{10}\left\{\prod_{i=1}^{n}T_{i}\right\}$$

Here, $T_{Mixture}$ is the transmittance of the mixture and $T_{i}$ the transmittance of element “i”. Transmittance is related to the absorption, expressed as a percentage, by the following equation:

(3) $$T_{i}=\;\left(1-\frac{{Abs}_{i}}{100}\right)$$

Here, ${Abs}_{i}$ is the absorption of element “i”. The absorption spectrum of a mixture is then given by Equation (4), which includes wavelength and concentration dependencies.

(4) $${Abs}_{Mix}(\lambda ,C_{1}\ldots C_{n})=100\left\{1-\prod_{i=1}^{n}\left(1-\frac{{Abs}_{i}(\lambda ,C_{i})}{100}\right)\right\}$$

Synthetic spectra were obtained by considering the 75 experimental spectra of the CEM dilution ranges [24] and the experimental dilution range spectra of the ESKAPEE bacteria [26]. Table 5 summarizes the number of synthetic spectra generated for each type of bacterial contamination. Overall, 19,125 synthetic spectra were generated.

## 3. Results

The goal of this work is to demonstrate a method for detecting contamination during T-cell culture that could potentially be adapted into a sampling-free, real-time technique. The goal is to stop CAR T-cell production as early as possible, rather than identifying the cause of contamination. This section is divided into three parts. First, we demonstrate how the shape of the absorption spectra of contaminated CEM cultures changes as the contamination level increases and how this change can be used to detect contamination without sampling, and potentially in real time. Second, we present experimental results of CEM cultures contaminated with *E. coli* and demonstrate that white light spectroscopy can effectively detect bacterial contamination. In what follows, “CEM” stands for “CEM cells.” Finally, the expected results presented in the first part are compared to the actual experimental results obtained with contaminated cuvettes.

### 3.1. A Spectroscopic Marker of Bacterial Mammalian Cell Culture Contamination

#### 3.1.1. Definition of a “Warning Value” Indicating the Risk of Culture Contamination

**Figure 1:**
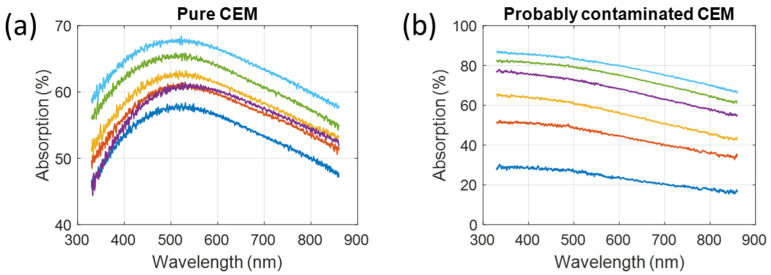
Absorption spectra shapes of (**a**) pure CEM cultures and (**b**) possibly bacteria-contaminated culture (extracted from data in [24]).

Previously, we reported how white light spectroscopy can be used to monitor the concentration of CEM cultures based on analyzing the shape of absorption spectra [24]. We demonstrated that the absorption spectra of T cells were approximately Gaussian. We also presented the shape of a CEM culture that was possibly contaminated with bacteria. These two cases had different absorption spectra shapes (Figure 1). The reason for this difference will be discussed in Section 4.5.

This current work is based on the hypothesis that the shapes of the absorption spectra of pure CEM cultures can be approximately described using a Gaussian function, while the shapes of contaminated cultures cannot. Therefore, when fitting spectra with a Gaussian-like function (Equation (5)), the R^2^ value of the fit should be high for pure cultures and low for contaminated ones.

(5) $${Abs}_{Warning}\left(\lambda \right)=a\cdot exp\left\{-{\left(\frac{\lambda -b}{c}\right)}^{2}\right\}+d$$

**Figure 2:**
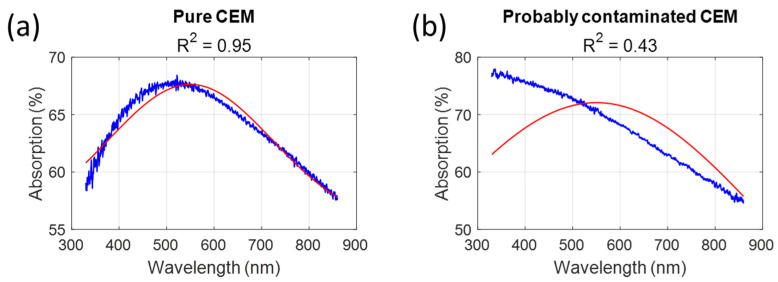
Examples of absorption spectra fitted with Equation (2). (**a**) Fitting pure CEM spectra yields R^2^ = 0.95. (**b**) Fitting a probably contaminated CEM yields R^2^ = 0.43.

In Equation (2), λ represents the wavelength; the other parameters are fitted. Equation (2) was used on some examples (Figure 2). The values of the R^2^ were clearly different.

However, precautions must be taken when fitting spectra. The parameters in Equation (2) cannot have any possible values. Bounds must be considered; otherwise, the fitting algorithm will always find a set of parameters that lead to high R^2^ values. Table 6 summarizes the bounds to be considered.

From there, a “warning value” can be deduced from the R^2^ value using Equation (6). The higher the warning value, the higher the risk of contamination.

(6) $$Warning=1-R^{2}$$

The spectra fitting with Equation (2) will be discussed in Section 4.5.

Indeed, the warning value is simply a descriptor that measures how Gaussian-like the absorption spectra are. It is called a “warning value” because it can trigger an alarm and stop the cell culture as soon as the spectra differ from their Gaussian-like shape due to contamination.

#### 3.1.2. Calculation of a Warning Threshold Above Which Contamination Is Detected

**Figure 3:**
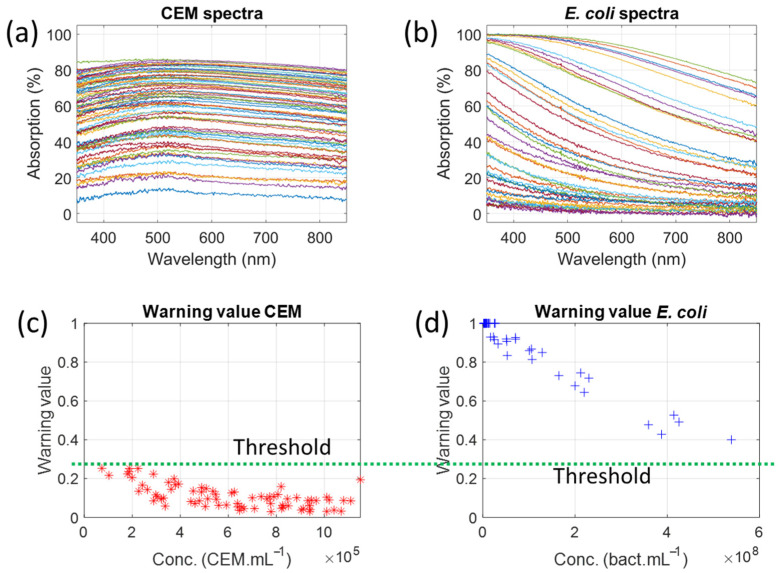
Warning values calculated with absorption spectra previously reported (red and blue ‘+’ for CEM and *E. coli* respectively, green dashed line: threshold value). (**a**) CEM dilution ranges: 7.33 × 10^4^ to 1.15 × 10^6^ CEM.mL^−1^ (from [24]). (**b**) *E. coli* dilution ranges: 1.69 × 10^6^ to 5.39 × 10^8^ bact.mL^−1^ (from [26]). (**c**) Warning values for CEM. (**d**) Warning values for *E. coli*.

Warning values were calculated with the CEM and the *E. coli* dilution ranges already reported [24,26] (Figure 3). The CEM concentrations varied from 7.33 × 10^4^ to 1.15 × 10^6^ CEM.mL^−1^ (Figure 3a) and the *E. coli* concentrations from 1.69 × 10^6^ to 5.39 × 10^8^ bact.mL^−1^ (Figure 3b).

Since the higher warning value for CEM is about 0.25 and the lower warning value for *E. coli* is approximately 0.4, a warning threshold must be chosen between these two values. To detect a possible contamination as early as possible, the warning threshold is set at 0.26 (Figure 3c,d), which is just above the maximum warning value for pure CEM. 0.26 (see Figure 3c,d), which is just above the maximum warning value for pure CEM. ROC (Receiver Operating Characteristic) curve analysis confirms that the optimal threshold is 0.25 (see Section 4.3). The 0.26 threshold is used to ensure that pure CEM cells are not considered contaminated.

#### 3.1.3. Determination of the Minimum Bacteria Concentration to Trigger a Contamination Alarm

As contamination develops, the warning value evolves from below the threshold (ideally 0) to above the threshold (up to 0.4 for *E. coli*). The minimum bacteria concentration at which the alarm is triggered depends on the CEM concentration. These calculations were performed using synthetic spectra derived from the experimental spectra shown in Figure 3. Equation (4) was used to calculate them as follows.

(7) $${Spec}_{Mix}^{Exp}(\lambda ,C_{CEM},C_{E.coli})=100\left\{1-\left(1-\frac{{Spec}_{CEM}^{Exp}(\lambda ,C_{CEM})}{100}\right)\left(1-\frac{{Spec}_{E.coli}^{Exp}(\lambda ,C_{E.coli})}{100}\right)\right\}$$

In Equation (7), $\;{Spec}_{CEM}^{Exp}\left(\lambda ,C_{CEM}\right)$ represents one of the experimental CEM spectra of Figure 3a, and ${Spec}_{E.coli}^{Exp}(\lambda ,C_{E.coli})$ represents one of the experimental *E. coli* spectra of Figure 3b.

**Figure 4:**
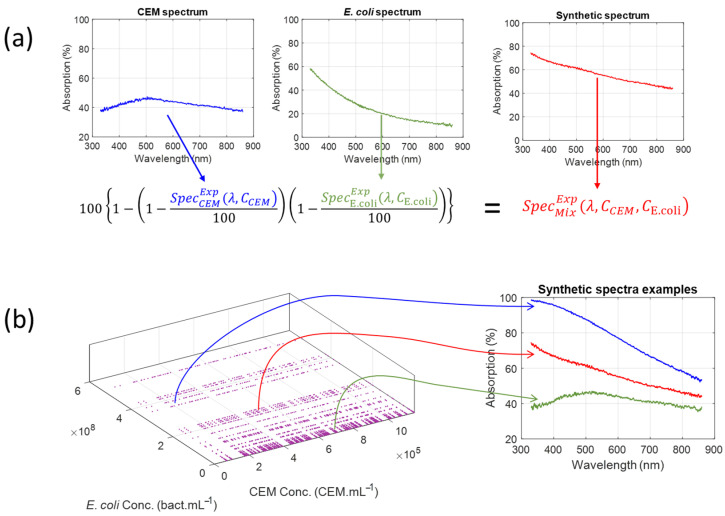
Synthetic spectra. (**a**) Illustration of the use of Equation (4) to generate synthetic spectra. (**b**) Examples of synthetic spectra generated from already published experimental data. Blue spectrum: 1.8 × 10^5^ CEM.mL^−1^ and 2 × 10^8^ *E. coli.*mL^−1^. Red spectrum: 3.21 × 10^5^ CEM.mL^−1^ and 7.09 × 10^7^ *E. coli.*mL^−1^. Green spectrum 3.74 × 10^5^ CEM.mL^−1^ and 1.69 × 10^6^ *E. coli.*mL^−1^.

Figure 4a illustrates the application of Equation (4) to generate a synthetic spectrum from the experimental spectra of 3.21 × 10^5^ CEM.mL^−1^ and 7.09 × 10^7^ *E. coli*.mL^−1^. Figure 4b shows synthetic spectra at various concentrations.

**Figure 5:**
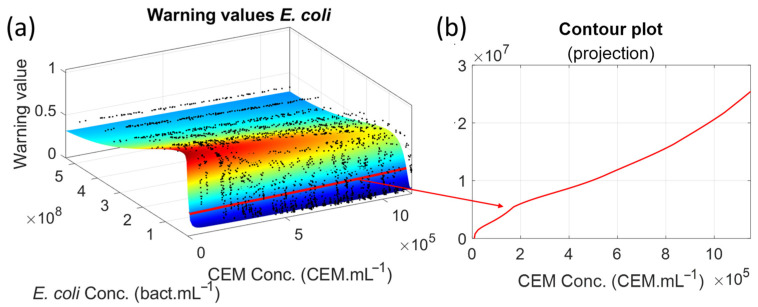
Warning values and minimum detectable *E. coli* concentration. (**a**) Warning values calculated from synthetic spectra and theoretical surface. (**b**) Minimum *E. coli* concentration for which an alarm can be triggered.

A total of 3000 synthetic spectra were generated for CEM contaminated with *E. coli*, and the warning value was computed for each of them (black dots in Figure 5a). The colored surface in Figure 5a corresponds to the theoretical warning values calculated using Equation (8), in which the theoretical CEM and *E. coli* functions reported in [24] and [26] respectively are used.

(8) $${Spec}_{Mix}^{Theo}(\lambda ,C_{CEM},C_{E.coli})=100\left\{1-\left(1-\frac{{Spec}_{CEM}^{Theo}(\lambda ,C_{CEM})}{100}\right)\left(1-\frac{{Spec}_{E.coli}^{Theo}(\lambda ,C_{E.coli})}{100}\right)\right\}$$

Theoretical shape functions for the spectra are recalled below together with tables providing the numerical values of the functions’ parameters for the CEM and *E. coli*.

Equation (9) describes the concentration-dependent evolution of the CEM absorption spectra. Refer to [24] for a complete description of this equation elaboration. It was found that the CEM spectra could efficiently be described using two Gaussian functions: one constant and the other concentration-dependent through its amplitude and width. The coefficients appearing in Equation (9) are given in Table 7.

(9) $${Spec}_{CEM}^{Theo}(\lambda ,C_{CEM})=100.\left(1-10^{-p1a1.C_{CEM}}\right).exp\left\{-{\left(\frac{\lambda -b_{1}}{p1c1.{C_{CEM}}^{p2c2}}\right)}^{2}\right\}+a_{2}.exp\left\{-{\left(\frac{\lambda -b_{2}}{c_{2}}\right)}^{2}\right\}$$

Equation (10) describes the concentration-dependent evolution of bacterial absorption spectra. Refer to [26] for a complete description of this equation’s development. All ESKAPEE bacteria were found to be described by the generic sigmoidal Equation (10). This function consists of an exponential baseline (the left term in Equation (10)) and a wavelength- and concentration-dependent sigmoid function (the right term in Equation (10)). The exponential baseline depends on concentration but not wavelength. The numerator of the sigmoid is a Gaussian function of the concentration, while the wavelength position of the sigmoid evolves exponentially with concentration. Note that, for mathematical simplicity and to account for bacterial concentration evolving over several orders of magnitude, the equation involves the log of bacterial concentration.

It was also demonstrated that each bacterium can be described by the same equation, albeit with its own set of parameters. The parameters for *E. coli* are listed in Table 8.

(10) $${Spec}_{E.coli}^{Theo}(\lambda ,{\tilde{C}}_{E.coli})=p1a1.exp\left(p2a1\left({\tilde{C}}_{E.coli}\right)\right)+\frac{p1a2.exp\left\{-{\left(\frac{{\tilde{C}}_{E.coli}-p2a2}{p3a2}\right)}^{2}\right\}}{5+exp\left\{10^{-2}\left(\lambda -p1a3+p2a3.exp\left(p3a3\left({\tilde{C}}_{E.coli}\right)\right)\right)\right\}}$$

With: ${\tilde{C}}_{E.coli}={log}_{10}(C_{E.coli})$.

**Table 8:** List of *E. coli* function parameters used in Equation (10).

Parameter	p1a1	p2a1	p1a2	p2a2	p3a2	p1a3	p2a3	p3a3
**Value**	6.47 × 10^−4^	1.31	360	7.9	1.08	36.9	7.21 × 10^−2^	1.04

Theoretical concentrations of species were considered to be between 5 × 10^4^ and 1.15 × 10^6^ CEM.mL^−1^ for CEM and between 1 and 5.39 × 10^8^ bact.mL^−1^ for *E. coli*. Theoretical CEM concentrations were not considered down to 0 because the theoretical function given in [24] was inefficient for very low mammalian cell concentrations (critical zone in Figure 5a) due to the large amplitude of the fixed Gaussian. Thus, the theoretical CEM function used to generate Figure 5 was slightly modified from that reported in [24] to better describe absorption spectra at low concentrations. Consideration about this modified function and the non-unicity of the CEM spectral function will be discussed in Section 4.2. Theoretical *E. coli* concentrations began at 1 bact.mL^−1^ because the theoretical *E. coli* function is expressed in terms of $\tilde{C}={log}_{10}(C)$ for which the concentration C = 0 is unacceptable.

**Figure 6:**
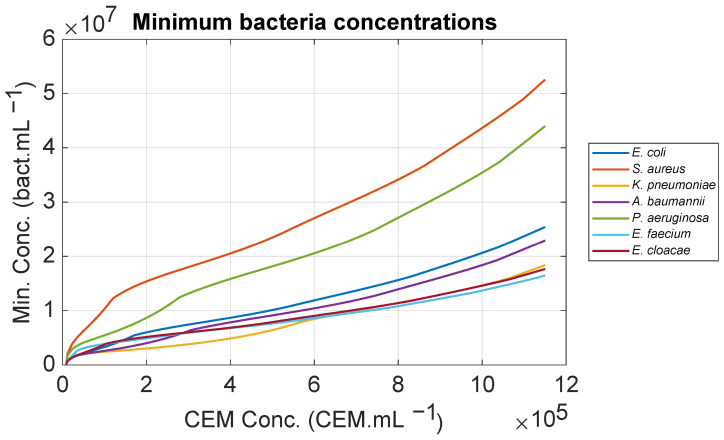
Minimum bacterial concentrations for which an alarm can be triggered as a function of the CEM concentration.

The contour plot shown as a red line was drawn for a Z-value of 0.26 corresponding to the warning threshold (Figure 5a). Projecting this contour plot onto the (X, Y) plane corresponds to the minimum *E. coli* concentration at which an alarm can be triggered (Figure 5b). The same calculations were conducted for all ESKAPEE bacteria as a function of CEM concentrations (Figure 6). One interesting finding would be determining the time required to reach these minimum concentrations. However, referring to the generation times of bacteria cultivated in their optimum culture media is not possible here, as this will be discussed in Section 4.4.

To our knowledge, this is the first time the concept of using white light spectroscopy to detect contamination has been proposed.

### 3.2. Experimental Validation

As mentioned in Section 2, six contamination experiments were conducted over three experimental sessions during which absorption spectra were regularly recorded for 11 h. For each session, a CEM control cuvette was prepared. In each session, four contaminated cuvettes were prepared (2 contaminated at 10^6^ bact.mL^−1^ and 2 at 10^2^ bact.mL^−1^). The following mainly presents the results obtained with contamination at 10^6^ bact.mL^−1^. Contamination at 10^2^ bact.mL^−1^ was below the inoculum threshold, which prevented bacterial proliferation.

#### 3.2.1. Species Concentration and Warning Value Evolution in the Control Cuvette During Session 1

**Figure 7:**
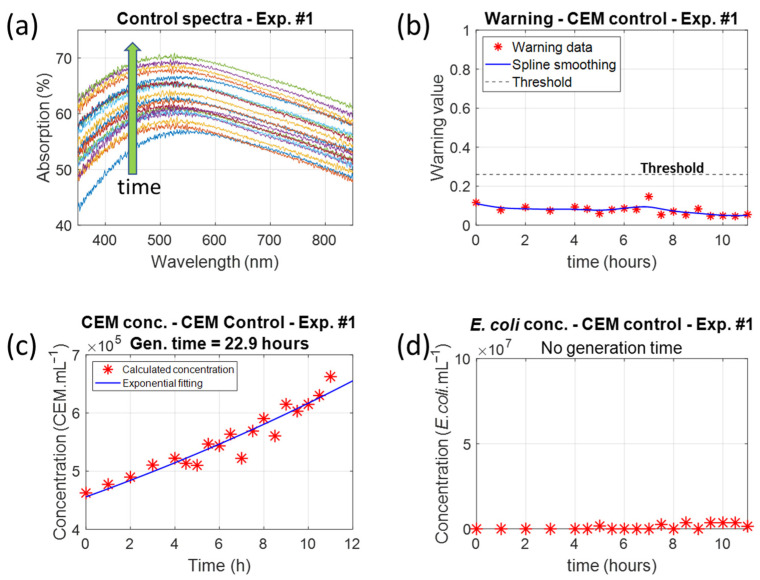
Data recorded with the control cuvette in Experiment 1. (**a**) Absorption spectrum evolution over time (the green arrow indicates an increase in time). (**b**) Corresponding warning values compared to the warning threshold. (**c**) The evolution of the CEM concentration shows a generation time of 22.9 h. (**d**) The evolution of the *E. coli* concentration shows no generation time.

To avoid lengthening this manuscript, we only present results obtained during session 1. Figure 7 shows the results obtained with the control cuvette. As expected, the absorption spectra maximum increased over time (Figure 7a). The corresponding warning values were always below the threshold (Figure 7b).

Species concentrations were measured using Equations (8)–(10) to fit the experimental absorption spectra (Figure 7c,d). The CEM generation time was calculated after fitting data in Figure 7c with an exponential function as cells grow exponentially. The generation time was approximately 23 h, which is consistent with the generally accepted value of about 24 h [28]. As expected, the *E. coli* concentration was not measurable in the control cuvette. Note that the CEM concentrations were not widely dispersed (disp. = 1.6 × 10^4^ CEM.mL^−1^, i.e., 2.8% at the center of the concentration range; see [24] for the dispersion calculation). This will be discussed in Section 4.1.

#### 3.2.2. Species Concentration and Warning Value Evolution in the Contaminated Cuvette During Session 1, Experiment 1

**Figure 8:**
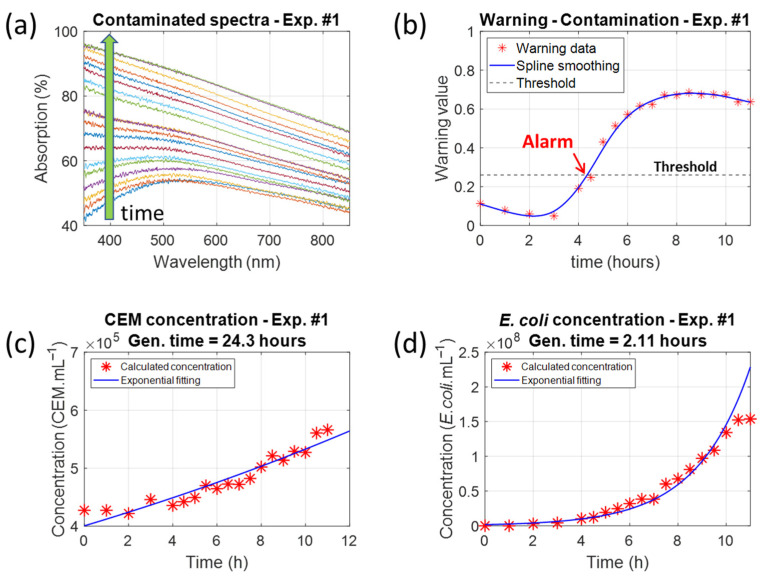
Data recorded with the contaminated cuvette in session 1, experiment 1. (**a**) Absorption spectra evolution over time (the green arrow shows increasing time). (**b**) Corresponding warning values compared to the warning threshold. (**c**) Evolution of the CEM concentration showing a generation time of 24.3 h. (**d**) Evolution of the *E. coli* concentration showing a generation time of 2.11 h.

CEM cuvettes with initial concentrations of approximately 4.5 × 10^5^ CEM.mL^−1^ were contaminated with *E. coli* concentrations of 1 × 10^6^ bact.mL^−1^. Figure 8 shows the results obtained for session 1, experiment 1.

The species concentrations were measured in the same way as the control cuvettes. The CEM concentration evolved with a generation time of 24.3 h. This was slightly higher than the generation time in the control cuvette due to bacteria, which slowed the CEM proliferation.

The *E. coli* bacteria proliferated with a generation time of 2.11 h. This was much higher than the commonly acknowledged 20 min because the bacteria were not in their optimal culture medium. Therefore, as discussed in Section 4.4, estimating the time to reach the warning threshold is not possible.

**Figure 9:**
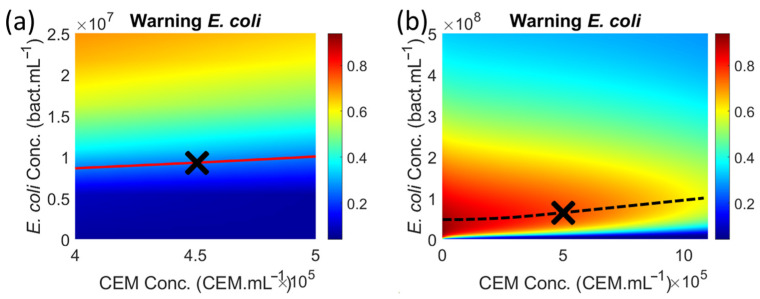
Approximate comparison between the expectations and the observations for experiment 1. (**a**) Species concentrations at the warning threshold (red line: threshold contour plot, black cross: species concentrations at threshold). (**b**) Species concentrations at maximum warning (black dotted line warning maxima, black cross: species concentrations at maximum warning in experiment 1). Zoomed top views of Figure 5a.

The warning threshold was reached after 4.35 h, when the CEM concentration was 4.5 × 10^5^ CEM.mL^−1^ and when the *E. coli* concentration was 1 × 10^7^ bact.mL^−1^ (Figure 9a, a zoomed top view of Figure 5a with removal of experimental data). The warning value reached a maximum after 8 h. This corresponded to a CEM concentration of approximately 5 × 10^5^ CEM.mL^−1^ and an *E. coli* concentration of approximately 7 × 10^7^ bact.mL^−1^ (Figure 9b). The minimum *E. coli* detectable concentration is then seven times lower than that measured for the maximum warning value. However, these numerical values are approximate. An accurate comparison between the expected results (Section 3.1) and the observed results (Section 3.2) will be presented in Section 3.3.

#### 3.2.3. Summary of All Experimental Results

**Figure 10:**
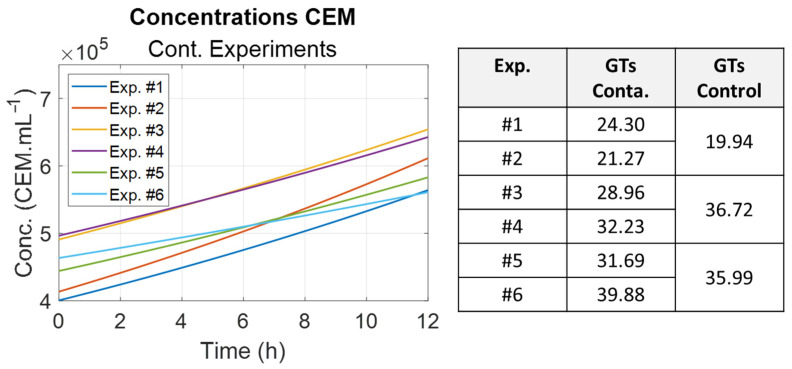
Summary of the CEM generation times for contaminated cuvettes. (**Left**): evolution of the CEM concentrations (smoothed data). (**Right**): generation times for each experiment with the generation times for the control cuvettes.

The evolution of CEM concentrations in contaminated cuvettes was compiled (Figure 10). The table on the right of the figure summarizes the generation times for each experiment, as well as generation times for the control cuvettes. The evolutions of the CEM concentrations were smoothed for clarity. In general, the generation times in the contaminated cuvettes were slightly longer than those in the control cuvettes. The longer generation time of the control cuvette in session 2 (experiments 3 and 4) was unexpected. In contaminated cuvettes, different mechanisms may occur. CEM growth may either be slowed down or stimulated, depending on their interaction with bacteria.

**Figure 11:**
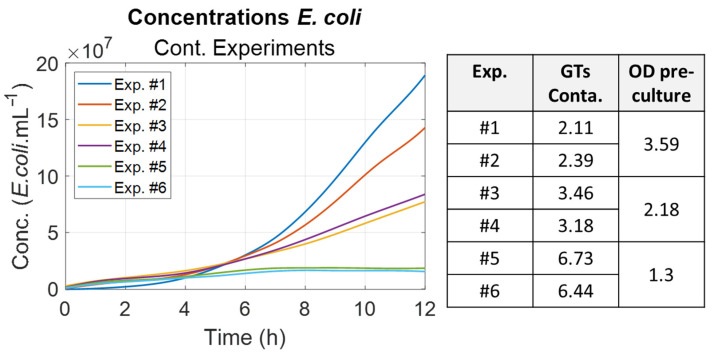
Summary of the *E. coli* generation times for contaminated cuvettes. (**Left**): evolution of the bacterial concentrations (smoothed data). (**Right**): generation times for each experiment with the corresponding optical densities (OD) of the bacteria pre-culture.

The same was performed for the *E. coli* concentrations (Figure 11). Generation times increased significantly between sessions 1 and 3. As expected, these times were considerably longer than the generation time of *E. coli* cultivated in their optimum medium. Optical densities (OD) were measured at the end of the bacteria pre-culture. An inverse correlation was observed, meaning that a low OD of pre-culture indicates that the bacteria are struggling to develop normally, as was directly observed in the contaminated cuvettes. This aspect will be discussed in Section 4.4.

**Figure 12:**
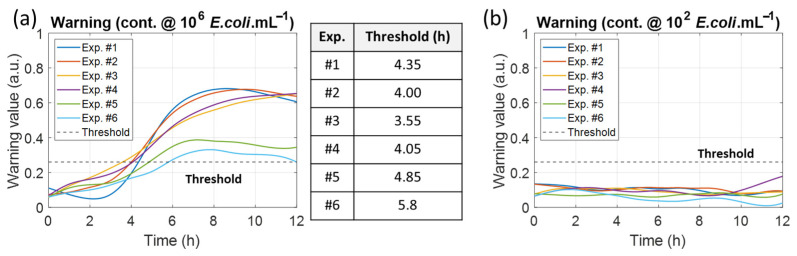
Evolution of the warning values in the contaminated cuvettes. (**a**) Warning values when the cuvettes were contaminated with 1 × 10^6^ *E. coli.*mL^−1^ with times when the warning threshold was reached. (**b**) Warning values when cuvettes were contaminated with 100 *E. coli.*mL^−1^ for comparison purposes.

Warning values were also compiled (Figure 12). The first observation was that there was no evident correlation between the time required to reach the threshold and generation time. The second observation is that, even when bacteria barely proliferated, the threshold was still reached, albeit much later. Finally, when contamination was very low (10^2^ bact.mL^−1^), the warning value was always below the threshold because the bacteria concentration was below the inoculum threshold, which prevented any bacterial proliferation.

### 3.3. Comparison Between Expected and Experimental Results

**Figure 13:**
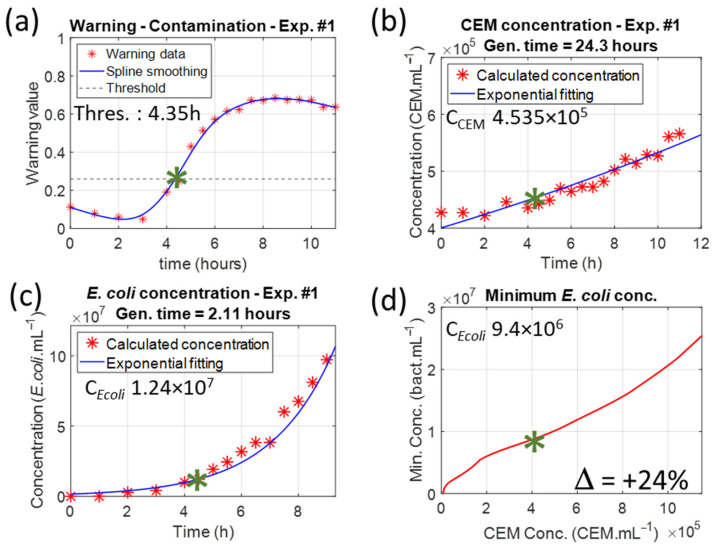
Comparison between experimental species concentrations and what was expected from synthetic data calculations. (**a**) Temporal evolution of the warning value during experiment 1. (**b**) Evolution of the CEM concentration. (**c**) Evolution of the *E. coli* concentration. (**d**) Comparison with expected concentrations values. Green stasr: warning value and species concentrations at threshold.

The species concentrations measured at the threshold were compared to the expected values based on the synthetic data calculations (Figure 13). For Experiment 1, the threshold was reached after 4.35 h (green star in Figure 13a). At that moment, the CEM concentration was 4.535 × 10^5^ CEM.mL^−1^ (green star in Figure 13b), and the *E. coli* concentration was 1.24 × 10^7^ bact.mL^−1^ (green star in Figure 13c). Looking at Figure 5b, which is reproduced in Figure 13d, the *E. coli* concentration should have been 9.4 × 10^6^ bact.mL^−1^ for 4.535 × 10^5^ CEM.mL^−1^ (green star in Figure 13d). The observed bacterial concentration was 24% higher than expected in this experiment. Similar overestimations were observed in all experiments as summarized in Table 9.

In fact, this logical overestimation is due to the presence of apoptotic and necrotic bodies generated during T cell growth that were not accounted for in synthetic computations. Using Equation (4) to fit absorption spectra allows one to calculate the CEM and bacterial concentrations simultaneously. This means that Equation (4) allows calculating concentrations of large particles (CEM) and small particles (bacteria) simultaneously. Since apoptotic and necrotic bodies are similar in size to bacteria, Equation (4) actually measures the concentration of CEM, bacteria, and apoptotic and necrotic bodies. The concentration of large particles actually corresponds to the concentration of CEM, while the concentration of small particles corresponds to the concentrations of bacteria, as well as the concentrations of apoptotic and necrotic bodies formed during cell multiplication. This explains the 23–28% overestimation reported in Table 9. However, the goal of this paper is to demonstrate the use of white light spectroscopy to detect contamination in real time without sampling, not to precisely identify or quantify contaminants. Indeed, detection is performed in real time during cell multiplication and bacterial proliferation without the need for quality control sampling (see the discussion in Section 4.4). Although the threshold is reached in about five hours, this can be considered real-time compared to the time most often required by conventional contamination detection methods (see Introduction).

### 3.4. Summary

This section presents a method for detecting bacterial contamination in mammalian cell cultures using white light spectroscopy. This method estimates the Gaussian-like shape of CEM absorption spectra to generate a warning value. When this value exceeds 0.26, an alarm can be triggered and production can be stopped immediately and early. This method can potentially be adapted for use with a real-time sampling-less device. To the best of our knowledge, this is the first time such a method has been proposed.

The next section will propose a discussion of the above results, the possible extension of the method to other contaminants, and the integration possibilities within a closed system.

## 4. Discussion

### 4.1. Accuracy of Species Concentrations Measurements

Figure 7c shows the calculated CEM concentration obtained by fitting the experimental spectra to Equation (8). This function enables the simultaneous calculation of bacteria and CEM concentrations, useful for monitoring the evolution of both populations in a co-culture. The dispersion was 1.6 × 10^4^ CEM.mL^−1^, representing about 2.8% accuracy. Since only CEM is present in the control cuvettes, one might think that fitting CEM spectra with an equation that calculates two different concentrations would reduce the accuracy of both. However, fitting the spectra shown in Figure 7a with Equation (9) (the spectra shape of pure CEM) results in a dispersion of 1.5 × 10^4^ CEM.mL^−1^, or 2.7%. Therefore, using the “mixture” function (Equation (8)) did not reduce the measurement accuracy.

Previously published studies reported measurement accuracies of 9% for T-cell lines and 10% for primary T-cells [24,27]. These values corresponded to the accuracies obtained for dilution ranges where spectroscopy cuvettes were changed at each measurement. These basic plastic cuvettes show dispersion of about 5%. However, when conducting monitoring experiments in which the cuvette is used throughout, accuracies of about 3% were obtained [24,26]. The same high accuracy was achieved in the present study.

Indeed, the “mixture” function was only used to monitor both species concentrations during the contamination experiments. Under normal circumstances and in an automated device (see below), the CEM function would be used to calculate the warning value and stop production early. After production is stopped, identification of bacterial contamination or other causes of poor-quality culture would be performed. At this stage, conventional identification and enumeration methods would be used.

### 4.2. Modified CEM Function and Non-Unicity of Functions Describing Absorption Spectra Shapes

Here, we consider the equation describing shapes of the CEM absorption spectra. The same remark holds for the shape functions of bacteria.

The function describing the CEM spectrum shape and its evolution with concentration was originally determined in two steps. First, a set of sequential fittings was used to find the general form of the CEM shape function. Then, a minimization algorithm was used to calculate the final function parameters [24]. The function consists of a concentration-dependent Gaussian plus a fixed Gaussian acting as a baseline, which is centered in the near-infrared region (936.1 nm) with a relatively large amplitude (12.21%).

**Figure 14:**
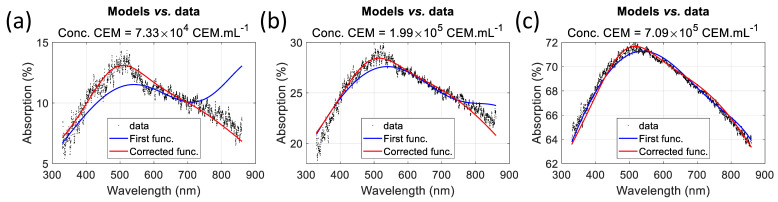
Fitting CEM spectra with the first and corrected functions. (**a**) At a low concentration: 7.33 × 10^4^ CEM.mL^−1^. (**b**) At an average concentration: 1.99 × 10^5^ CEM.mL^−1^. (**c**) At a high concentration: 7.09 × 10^5^ CEM.mL^−1^.

Using this function in Section 3.1.3 was not ideal for low CEM concentrations because the large amplitude of the fixed Gaussian resulted in a high warning value (low R^2^) even with very low bacterial concentrations. We then calculated a new set of parameters using a global fitting algorithm (3D fitting in MATLAB™), limiting the possible amplitude of the fixed Gaussian. This was not possible with other methods because the algorithms do not allow it. This method produced the parameters listed in Table 7, which allowed us to prove the concept of a warning value for low CEM concentrations, i.e., at the beginning of CAR-T cell production. We compared the fittings obtained using the initial and modified functions for very low, average, and high CEM concentrations (Figure 14). The influence of the large amplitude of the fixed Gaussian in the initial function is clearly visible at low concentrations (Figure 14b) and gradually diminishes as the CEM concentration increases.

**Figure 15:**
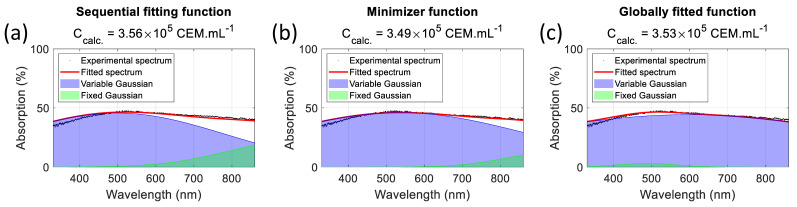
Determination of the CEM concentration and global dispersion using different sets of parameters. (**a**) Using sequential fitting, (**b**) using a minimization algorithm after the sequential fitting, and (**c**) using a global fitting algorithm.

Therefore, there is no unique set of parameters that can accurately determine the CEM concentration. Figure 15 shows examples of spectra fitting using various sets of parameters. Table 10 summarizes the method used to calculate the parameters, their values in each case, and the dispersion obtained over the 75 spectra of the dilution ranges used in [24]. The “sequential fitting” and “minimization algorithm” methods were published earlier and produce the “approximated” and “final” parameters [24]. The “globally fitted function” method is used in the present paper. Regardless of the method, the spectra are perfectly fitted. However, the characteristics of the fixed Gaussian differ. Using sequential fitting (Figure 15a), the fixed Gaussian is centered in the infrared region at 976.9 nm and is 253.9 nm wide. The same is true for the minimization algorithm with a fixed Gaussian centered at 936.1 nm and 177.2 nm wide (Figure 15b). As the concentration decreases, the fixed Gaussian becomes predominant, and the spectra cannot be efficiently fitted because of the infrared contribution (Figure 14a). Using the globally fitted function yields a fixed Gaussian centered at 487.2 nm and 107.5 nm wide (Figure 15c). Fitting spectra with this function is now efficient, even for absorptions below 15%, because the infrared component no longer exists (Figure 14a).

The CEM concentration of the experimental spectrum was measured using an automatic cell counter. 3.51 × 10^5^ CEM.mL^−1^. The titles of the subfigures in Figure 15 indicate the concentrations measured using the corresponding methods. The respective errors were +1.4%, −0.5%, and +0.5%, showing that the functions are equivalent in terms of concentration measurement accuracy.

In summary, the functions describing shapes of absorption spectra are not unique and can be adapted to each specific study.

### 4.3. Warning Threshold and Comparison with Statistical Species Classification

**Figure 16:**
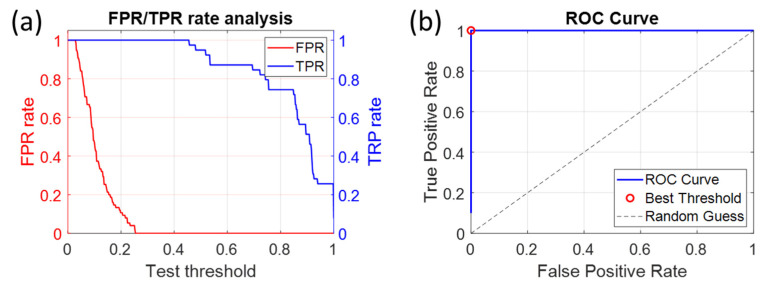
ROC curve analysis. (**a**) False positive rate (FPR in red) and true positive rate (TPR in blue) performed with CEM and *E. coli* data. (**b**) Corresponding ROC curve confirming an ideal warning threshold at 0.25.

The warning threshold was set at 0.26, which is just above the maximum warning value for pure CEM (0.25). A Receiver Operating Characteristic (ROC) curve analysis confirmed the mathematical warning threshold of 0.25 (Figure 16). Figure 16a shows the evolution of the false and true positive rates (FPR and TPR), and Figure 16b shows the ROC curve and the optimal warning threshold. The perfect shape of the ROC curve is due to the fact that the two populations (CEM and bacteria) are perfectly separated. The threshold of 0.26 was used to ensure that pure CEM is not considered contaminated. Figure 16 was obtained using CEM and *E. coli* data. The threshold value is the same for other ESKAPEE bacteria because the warning threshold is mainly governed by the warning values of CEM, and all ESKAPEE bacteria behave similarly spectrally [26].

A convenient and descriptive way to describe the shape of an absorption spectrum is to use principal component analysis (PCA, [26]). Furthermore, linear or quadratic discriminant analysis (LDA or QDA) can be applied to separate spectra corresponding to different species.

**Figure 17:**
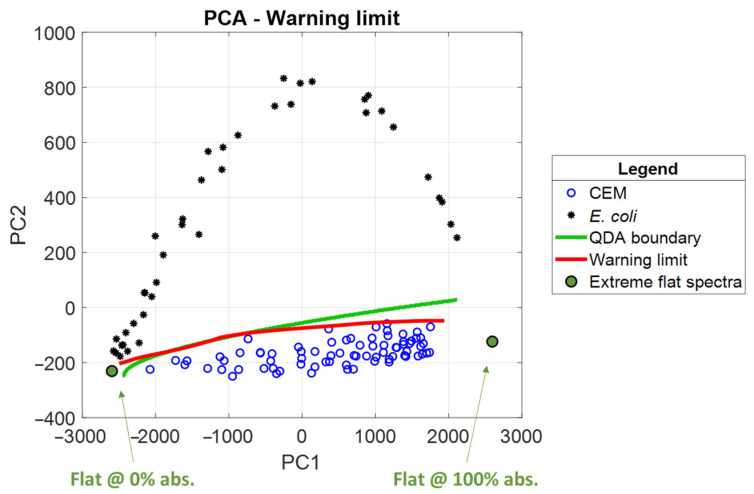
Principal component analysis and quadratic discriminant analysis applied to CEM and *E. coli* spectra (black stars and blue circles). The red line corresponds to the warning threshold and the green line to QDA. Green disks represent extreme absorption spectra cases. Increasing concentration from left to right.

This was applied to the CEM and *E. coli* dilution ranges (Figure 17). Only the first two principal components were considered, as they represent 99.8% of the information contained in the spectra. Black stars and blue circles represent the *E. coli* and CEM spectra, respectively. Increasing species concentrations are shown from left to right in the figure. Green disks represent extreme spectral shapes: flat spectra at 0% absorption for cuvettes filled with culture medium only (disk on the left) and flat spectra at 100% absorption for cuvettes filled with extremely high concentrations of either species. The red line in Figure 17 represents synthetic spectra corresponding to the contour line in Figure 5a, which represents the warning threshold. The green line represents the CEM-*E. coli* separation frontier obtained by QDA. Only QDA is represented here because it produces a diagonal confusion chart, while LDA does not.

Our method is equivalent to or better than QDA for medium and high concentrations even if QDA is slightly better for very low concentrations. Therefore, our method is quite competitive with the conventional method of species separation using QDA. Indeed, QDA determines the optimal separation between two classes. Our method determines the outer frontier of the CEM region.

### 4.4. Concerning the Time to Trigger the Alarm and Bacteria Generation Times

Determining a minimum bacterial concentration makes it possible to trigger an alarm (Figure 6). We acknowledge that the pertinent question is “How long does it take to trigger the alarm after possible contamination?”.

To answer this question, ESKAPEE bacteria would need to be cultivated in a mammalian cell culture medium to determine their generation times under these conditions. While this could be performed, the added value would be minimal. A better approach would be to perform these experiments in co-culture with mammalian cells at various initial concentrations. This would allow considering competition between species during co-culture experiments. However, this method would not allow determining the lag time of bacteria in non-optimum culture media.

The six experiments presented here showed generation times of several hours instead of the usual tens of minutes for ESKAPEE bacteria. In these conditions, the time required to reach the warning thresholds was about 5 h. This time should be compared to the 7–10-day duration of the CAR-T cell production expansion phase. We recall that the goal of our work is to detect contamination during production as early as possible without sampling, thus avoiding the added risk of contamination due to quality control itself (see below for more information on the integration and real-time potential of the method). Five hours appears to be nearly real-time compared to current methods, which consist of sampling and cultivating the sample prior to measurement and potential detection. Our method consists of real-time measurements and direct monitoring of cultures and contaminations that could occur in cuvettes, rather than the detection of priming contamination.

As described in the introduction, most traditional techniques (e.g., PCR, ELISA, and culture) require sampling and cultivation prior to measurement. This process takes at least 18–20 h, or one night, to obtain sufficient bacteria for detection [3,4]. In addition to this incubation time, the time required for measurement and analysis should also be considered. Furthermore, the BacT/ALERT 3D system requires up to five or six days to confirm the absence of microbial growth [20]. However, this equipment is used to certify the absence of contamination. Our method simply triggers an alarm as soon as contamination is detectable during the expansion phase.

It was noted that the *E. coli* generation times were different and increased between experimental sessions. They were inversely proportional to the optical density measured for the bacteria preculture used for contamination (Figure 11). Indeed, it is commonly observed that a bacteria’s ability to grow is directly related to the preculture optical density [29].

However, the warning threshold was reached even with very long generation times. This efficiency is due to the chosen threshold value of 0.26, which is just above the warning value for pure CEM, and to the contamination concentration of 10^6^ *E. coli* mL^−1^. When the contamination was at 10^2^ *E. coli* mL^−1^, the warning threshold was never reached, and no realistic *E. coli* concentration could be measured using Equation (8). It was due to an *E. coli* concentration below the inoculum threshold in this non-optimum culture medium. Therefore, the method described here cannot detect contamination at low concentrations of contaminants. Nevertheless, the method remains valid in the context of CAR-T cell production mentioned above because it only addresses the expansion phase of the fabrication process for which automated, sampling-less controls are desired. The main points are listed below.

- Contamination may occur during the expansion phase.
- The development of contamination depends on the culture medium used for cell culture, which may be more or less favorable to the contaminating microorganism.
- Our goal is to detect this contamination without sampling to provide a rapid response and to stop production as early as possible.
- We can only detect contamination if it has developed enough to be detected by our method. In our experiments, inoculation at 10^2^ bact.mL^−1^ was insufficient for contamination to develop in the CEM-C1 culture medium. An inoculation of 10^6^ bact.mL^−1^ was sufficient for the contamination to develop and for us to prove the concept of our method.
- Inoculation at concentrations too low for contamination to develop cannot be detected by the closed system and real-time technique we propose, but contamination does exist. In any case, detecting contamination is mandatory and must be performed at the end of ATMP production using any available sensitive method. At this stage of production, the need for rapid detection without sampling is no longer an issue. This ultimate control is not the subject of our study.

Another aspect concerns the generation of small particles, such as apoptotic and necrotic bodies and vesicles, when cells grow normally (see Section 3.3). The CEM model was originally established using cells after centrifugation and resuspension in PBS [24]. Consequently, there were no small particles in the spectra shown in Figure 3. The contamination experiments presented in this paper were performed directly in the culture medium without centrifugation. Consequently, small particles were not removed before recording the spectra. Although small particles are produced in limited quantities, they slightly modify the shape of the cells’ absorption spectra, though not as much as bacterial contamination. This will slightly increase the warning values and, consequently, the warning threshold. Contamination will still be detectable, but it will take longer to reach the threshold and trigger an alarm, and the minimum detectable bacteria concentration will increase. For practical applications, the warning values and threshold must be determined.

### 4.5. Origin of the Contaminated Spectra Shape Evolution and General Use of the Warning Function

From an optical perspective, the shape of an absorption spectrum depends on the intrinsic absorption and the size of the particles interacting with light. For large particles, such as CEM, light is primarily absorbed by the intracellular constituents and propagates in a straightforward manner. For CEM, this results in a Gaussian-like absorption spectrum, which is described by Equation (9) (Figure 1a). For small particles (bacteria, for example), light does not propagate in a straight line but rather undergoes diffusion. For particles of bacteria size, diffusion evolves according to 1/λ, producing characteristic absorption spectra described by Equation (10) [26]. During contamination, the absorption spectra evolve from a Gaussian-like shape to a shape deformed by the 1/λ diffusion efficiency (Figure 8a). We calculated these equations describing the shapes of both species using dilution ranges of pure particles, i.e., CEM resuspended in PBS and bacteria in their culture medium.

The mixture function (Equations (7) and (8)) enables the determination of the concentrations of different suspended species. Optically, this means that the mixture function enables the simultaneous calculation of the concentrations of large and small particles. As CEM cells grow, they produce new cells and small particles (e.g., vesicles, debris), and some cells die, producing other types of small particles (e.g., necrotic or apoptotic bodies). Small particles are produced more abundantly when CEM multiplication is suboptimal, such as in the case of contamination. Since the mixture function only accounts for large and small particles, the measured CEM concentration is correct, but the measured bacterial concentration is overestimated due to the presence of small particles produced by the CEM. This is why the *E. coli* concentration was overestimated in Section 3.3. Since the initial CEM and *E. coli* concentrations were similar for all experiments, the overestimation remained consistent across all experiments, as summarized in Table 9.

When used to fit absorption spectra, the warning function (Equation (5)) produces an R^2^ value, which is a measure of the Gaussian-like aspect of the absorption spectra. It is a general Gaussian-like marker, regardless of the reasons spectra deviate from a Gaussian shape. This warning function was used to correlate the Gaussian aspect of the absorption spectra of dying cells to cell viability, although in a slightly different manner. In fact, cells produce small particles when they die [30]. Indeed, this Gaussian-like marker is a more general indicator of abnormal cell culture, whether due to contamination or abnormal cell mortality.

Regarding CAR-T cell production, the warning function and the corresponding R^2^ value serve as a “quality” marker that triggers an alarm to stop production as early as possible when the expansion phase is abnormal.

### 4.6. Extension of the Method to Other Bacteria or Contaminants

This method can be applied to other types of contaminants. The only restriction is that the absorption spectra of the contaminants must differ significantly from that of CEM in order to compute a high warning value, which is often related to the contaminants’ size.

ESKAPEE bacteria range in size from approximately 0.5 to 2 µm. Diffusion is predominant for them, and the method can easily be applied, as shown in Figure 6. The position of ESKAPEE bacteria in the PCA representation has been reported previously [26]. They are all situated in the same area as *E. coli*, i.e., far from the threshold limit in the PCA representation.

**Figure 18:**
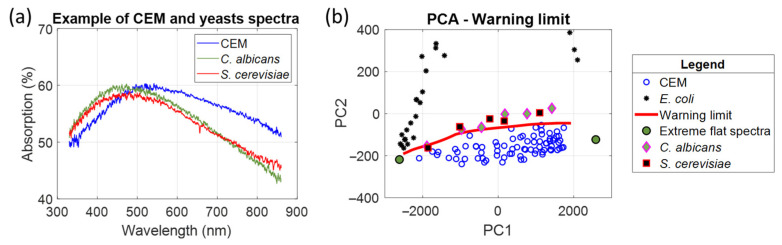
Comparison between CEM and yeasts. (**a**) Absorption spectra of CEM compared to *C. albicans* and *S. cerevisiae*. (**b**) PCA representation including these yeasts.

Yeasts are similar in size to CEM. Their length ranges from 2–3 µm to 20–50 µm, and their diameter ranges from 1 to 10 µm [31]. Only two yeasts were tested. Several absorption spectra of *Candida albicans* (5–6 µm) and *Saccharomyces cerevisiae* (5.5–7.5 µm) were recorded and included in the PCA representation (Figure 18).

Although they are different, their spectra are similar to those of CEM (Figure 18a). However, the shape of their spectra above 600 nm suggests that these yeasts diffuse light slightly. These similarities with CEM spectra are clearly visible in the PCA representation (Figure 18b). Nevertheless, at certain concentrations, they exceed the threshold frontier (1.1 × 10^6^ cells mL^−1^ for *C. albicans* and 0.4 × 10^6^ cells mL^−1^ for *S. cerevisiae*). This demonstrates that this type of contamination can be detected, albeit not as easily as bacterial contamination. However, the wide disparity in yeast sizes makes it difficult to generalize the ability to detect contamination in all yeasts.

Fungal contamination is probably much more difficult to detect. Most microscopic fungi are 2 to 10 µm in diameter and millimeters in length, though some, like *Fungal hyphae*, are only 5 to 50 µm in length [32]. Without performing absorption spectra measurements, it is difficult to estimate the detectability of contamination by such microorganisms.

In conclusion, specific experiments must be conducted for each type of non-bacterial contaminant. Contamination can only be detected if the absorption spectra of the contaminants differ sufficiently from those of mammalian cells to exceed the alert threshold.

### 4.7. Integration Possibilities and First Real-Time Experiment

**Figure 19:**
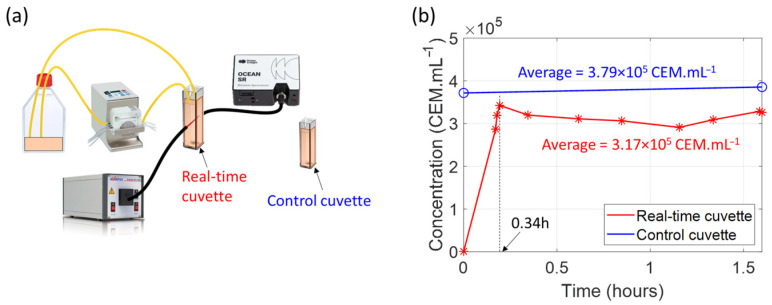
First demonstration of a real-time and sampling-free experiment. (**a**) Schematic setup [23]. (**b**) Real-time and sampling-less measurements.

The integration possibilities of this method and other proofs of concept on different subjects have been discussed extensively [22,23,27]. For this study, we began integrating a real-time, closed-loop system based on a previously published schematic setup (Figure 19a, [23]). In a recent preliminary experiment, we replaced the bioreactor with a culture flask containing a CEM suspension at a concentration of 3.8 × 10^5^ CEM.mL^−1^ and an additional control cuvette containing the same concentration of CEM. Fluid was driven using a peristaltic pump. Spectroscopically measured concentrations were recorded regularly during the 1.5 h experiment at room temperature. At the beginning of the experiment, the spectroscopy cuvette was filled with culture medium only for the reference measurement (Figure 19b).

As mentioned above, at t = 0, the spectroscopy cuvette is filled only with culture medium, and the measured concentration is 0.

After turning on the peristaltic pump, the medium in the spectroscopy cuvette is progressively replaced by the cell suspension. The measured concentration stabilizes after 0.34 h. The concentration in the spectroscopy cuvette remained relatively constant throughout the experiment, demonstrating the safety of using a peristaltic pump for cells.

The concentration in the spectroscopy cuvette is lower than the concentration in the control cuvette. This is due to the slight dilution of the culture flask’s contents when the spectroscopy cuvette’s culture medium is mixed in. The culture flask originally contained 20 mL of cell suspension, and the spectroscopy cuvette was filled with 3 mL of culture medium. The initial concentration of 3.8 × 10^5^ CEM.mL^−1^ decreases by a factor of 1.19 during the experiment, while the total volume increases from 20 mL to 23 mL, representing an increase of 1.15, which is consistent with the change in concentration.

Although contamination has not yet been tested, these preliminary results demonstrate the feasibility of a real-time, sampling-less monitoring device based on white light spectroscopy. In the future, the automated experiment shown above will enable us to conduct a large number of experiments under various culture conditions and with different contaminants. This will allow us to estimate the reliability of our white light spectroscopy method.

### 4.8. Position of Our Studies Compared to Others

The method we developed in recent years, based on white light spectroscopy, has allowed us to measure cell and bacterial concentrations, monitor growth, and determine concentrations of both cells and bacteria in mixtures [24,26]. The present study highlights another application: detecting bacterial contamination in mammalian cell cultures in real time based on defining a warning value and corresponding warning threshold. Below, we present the advantages and disadvantages of this technique compared to those described in the literature.

Measurements are performed in a large volume, which leads to a high level of accuracy in determining cell and bacterial concentrations, even during growth. This is superior to other techniques that require sampling and/or working with a small volume, which could be poorly representative of the bioreactor’s contents. This large measurement volume is acceptable in a closed system because no suspension volume is sacrificed. This is not the case in conventional laboratory practice. The large volume can be obtained using a derivation directly in the bioreactor with a reflection probe.

Classical detection techniques based on DNA [4] or ligand immobilization [12,13] often require additional time for experiments and/or bacterial cultivation. Our system enables the detection of cell culture contamination in nearly real time. Indeed, the experiments presented here were performed in spectroscopic cuvettes, but preliminary experiments demonstrate the feasibility of real-time detection in cell flasks.

Most techniques used for bacterial detection only reveal the presence of bacteria and do not provide information about the state of mammalian cells. Very few studies have employed simultaneous detection methods that combine Raman spectroscopy and advanced signal processing [33]. Our system is powerful because it can measure the concentration of both species in a solution without sampling and trigger an alarm to stop cell cultivation as quickly as possible.

In recent years, bacterial detection has improved with the help of fluorescence, such as with flow cytometry [34], or other techniques [10]. One advantage is that our system allows for direct, label-free detection of bacteria, as opposed to indirect measurements of metabolites, ATP, and gases secreted or emitted by bacteria [35,36]. Moreover, this simple method, based on the old technique of white light spectroscopy, can be easily transferred and does not require complex technologies, such as microfluidics or mass spectrometry.

The methods used for bacterial detection can identify the bacteria responsible for contamination. However, this does not correspond to the goal of the present study, which is to detect contamination in mammalian cell cultures, since most of the time, the only necessary application is to stop the culture. Other methods involving immobilization on ligands (e.g., ELISA, SPR, and QCM) require prior knowledge of the bacteria [14,15,16]. In contrast, our system can rapidly detect bacteria regardless of their origin. All of the bacteria tested displayed similar spectra profiles, which were completely different from those of mammalian cells [26], and generated different spectra while growing within cell media.

Developments in the health and agri-food industries require the detection of bacteria at very low concentrations. Our experiments were performed with a relatively high bacterial concentration for inoculation, i.e., 10^6^ bact.mL^−1^. Depending on the bacteria and/or cell media, this concentration can be reached within a day if there are enough bacteria present to develop. Specific experiments can determine the minimum bacterial inoculation that leads to detectable contamination. In all cases, 10^6^ bact.mL^−1^ is acceptable for monitoring CAR-T cell production during the expansion phase. To our knowledge, this is the first study to allow real-time determination of both cell and bacterial concentrations. More importantly, it is the first proposal of a real-time warning method to stop CAR-T cell production as soon as contamination occurs.

More generally, CAR-T cell therapy is a groundbreaking approach to treating currently incurable diseases. While this technology shows great promise, it is difficult to predict which conditions will respond best or how many people will benefit. Currently, several CAR-T cell therapies are available, but they are costly due to the complex manufacturing and administration processes. While these therapies have demonstrated effectiveness in treating various hematologic malignancies, they also incur substantial additional expenses, particularly those associated with hospital stays and managing adverse effects, such as cytokine release syndrome (CRS). Consequently, the total cost per patient can approach or even exceed $1 million [37,38].

Introducing automation into CAR-T cell production could play a key role in reducing expenses. Automation decreases reliance on specialized personnel and minimizes human error. It has the potential to streamline manufacturing, improve reproducibility, and increase access. Automation could also accelerate production timelines, enabling patients to begin therapy sooner and potentially reducing the need for interim treatments. Ultimately, these improvements could lessen the strain on healthcare systems and improve the affordability and accessibility of CAR-T cell therapies.

## 5. Conclusions

We presented here a white light spectroscopy method for detecting bacterial contamination in T cell cultures. It is based on the difference in absorption spectra shape between pure T cells and bacteria due to the difference in light interaction with small and large particles. We proposed a warning function to analyze the absorption spectra of potentially contaminated cultures. This enabled defining a warning value based on the degree of resemblance between the shape of the suspension absorption spectra and the Gaussian distribution. Contamination is detected when the warning value reaches a threshold.

We presented numerical proof of concept using synthetic spectra generated from actual experimental spectra of T cells and ESKAPEE group bacteria, employing the law of optical density additivity. Fitting the spectra with the warning function yielded an R^2^ value inversely proportional to the proposed warning value. A warning threshold of 0.26 was defined. The minimum bacterial concentrations required to trigger a contamination alarm were calculated for bacteria of the ESKAPEE group.

Experiments conducted with CEM-C1 cells (a T cell line) and *E. coli* bacteria demonstrated the efficiency of contamination detection. The warning threshold was reached between 3.55 and 5.8 h after inoculation at 10^6^ bact.mL^−1^, corresponding to *E. coli* concentrations between 1.24 × 10^7^ and 1.48 × 10^7^ bact.mL^−1^. These values were slightly higher than the calculated theoretical values (0.94 × 10^7^ and 1.08 × 10^7^ bact.mL^−1^), due to the generation of apoptotic and necrotic bodies during T cell growth, which was not taken into account in synthetic computations. When the threshold was reached, CEM concentrations were between 4.34 × 10^5^ and 5.08 × 10^5^ CEM.mL^−1^. For CEM concentration at 5 × 10^5^ CEM.mL^−1^, the theoretical minimum detectable bacteria concentration was approximately 0.9 × 10^7^ bact.mL^−1^ for all ESKAPEE bacteria except for *P. aeruginosa* (2 × 10^7^ bact.mL^−1^) and *S. aureus* (2.7 × 10^7^ bact.mL^−1^). PCA analysis confirmed that the method is more effective than QDA-based techniques. A discussion of the presented results was proposed and included a demonstration of the first attempt at real-time, sampling-less measurements.

Using white light spectroscopy makes it possible to reduce unnecessary sampling and the time required to stop ATMP production when contamination is detected. This should lower the cost of these promising medicines and make them more accessible.

## Figures and Tables

**Table 1:** Culture media for cell culture.

Reagent	Supplier (Country)	Reference
RPMI-1640 phenol red free	PAN-Biotech^®^, Aidenbach, Germany	P04-16515
HEPES	PAN Biotech^®^, Aidenbach, Germany	P05-01500
FBS	Fischer Scientific^®^, Illkirch, France	10270-106
Penicillin/streptomycin	TransGen Biotech^®^, Beijing, China	FG101-01

**Table 2:** Culture media and buffer for bacteria culture.

Reagent	Supplier (Country)	Reference
LB Broth	Difco™, Fisher Scientific™, Illkirch, France	241420
TSB	Oxoid™, Fisher Scientific™, Illkirch, France	CM1065T
Petri dishes	Starstedt, Numbrecht, Germany	82.1184.500
NaCl 0.85%	Dutscher, Bernolsheim, France	994004

**Table 3:** Equipment, suppliers, and references for spectral measurements.

Equipment	Supplier (Country)	Reference
White light source	Avantes^®^ (The Netherlands, Apeldoorn, supplier France)	Avalight-DH-S-BAL
Cuvette holder	Avantes^®^ (The Netherlands, Apeldoorn, supplier France)	CUV-UV/VIS
Optical fibers	Thorlabs, USA, supplier Maisons Laffitte, France	M25L01
Spectroscopy cuvettes	Sigma, Saint Louis, MO, USA	CO793-100EA
Spectrometer	Ocean Optics (USA, supplier Sergy, France)	USB 4000 UV-VIS-ES
OceanView software	Ocean Inside (USA, supplier Sergy, France)	Version 2.0.8

**Table 4:** Number of spectra acquired during the 6 experiments.

	Session 1	Session 2	Session 3
	N_control-1_ = 19	N_control-2_ = 12	N_control-3_ = 12
	N_exp1_ = 19	N_exp3_ = 12	N_exp5_ = 12
	N_exp2_ = 19	N_exp4_ = 12	N_exp6_ = 12
Total	57	36	36

**Table 5:** Number of synthetic spectra per bacteria type.

*Bacteria*	*Escherichia* *coli*	*Staphylococcus* *aureus*	*Klebsiella* *pneumoniae*	*Acinetobacter* *baumannii*	*Pseudomonas* *aeruginosa*	*Enterococcus* *faecium*	*Enterobacter* *cloacae*
**Number of spectra**	3000	2625	2625	2700	2625	2625	2925

**Table 6:** Bounds to be considered when fitting spectra.

Parameter	A	b	c	d
**Lower bound**	0	550	0	0
**Upper bound**	200	700	500	200

**Table 7:** List of CEM function parameters used in Equation (9).

Parameter	p1a1	b1	p1c1	p2c1	a2	b2	c2
**Value**	7.20 × 10^−7^	596.0	9.25	0.34	3.05	487.2	107.5

**Table 9:** Bacterial concentration overestimations.

Exp.	Time to Threshold (h)	Measured CEM.mL^−1^ (×10^5^)	Measured bact.mL^−1^ (×10^7^)	Theoretical bact.mL^−1^ (×10^7^)	Overestimation (%)
**1**	4	4.53	1.24	0.94	24%
**2**	4.35	4.70	1.30	0.96	26%
**3**	3.55	4.34	1.48	1.07	28%
**4**	4.05	4.42	1.43	1.08	25%
**5**	4.85	4.96	1.34	1.01	25%
**6**	5.8	5.08	1.33	1.02	23%

**Table 10:** Methods and resulting parameters used to fit the CEM concentrations.

Parameter	p1a1	b1	p1c1	p2c1	a2	b2	c2
**Sequential fitting**	7.45 × 10^−7^	496.9	2.14	0.41	23.11	976.9	253.9
**Minimization**	7.67 × 10^−7^	533.7	6.32	0.34	12.21	936.1	177.2
**Global fitting**	7.20 × 10^−7^	596.0	9.25	0.34	3.05	487.2	107.5

## Data Availability

Research data are available on demand to the corresponding author.
